# Human Metapneumovirus and Severity of Respiratory Syncytial Virus Disease

**DOI:** 10.3201/eid1007.030983

**Published:** 2004-07

**Authors:** Isaac Lazar, Carla Weibel, James Dziura, David Ferguson, Marie L. Landry, Jeffrey S. Kahn

**Affiliations:** * Yale University School of Medicine, New Haven, Connecticut, USA

**Keywords:** human metapneumovirus, hMPV, HMPV, respiratory syncytial virus, RSV, co-infection, Pediatric Intensive Care Unit, PICU, dispatch

We screened 23 children with severe respiratory syncytial virus (RSV) disease and 23 children with mild RSV disease for human metapneumovirus (HMPV). Although HMPV was circulating in Connecticut, none of the 46 RSV-infected patients tested positive for HMPV. In our study population, HMPV did not contribute to the severity of RSV disease.

In the United States, 100,000 infants and young children are hospitalized each year with respiratory syncytial virus (RSV) bronchiolitis (1). Although the risk factors for severe RSV disease, such as prematurity and bronchopulmonary dysplasia, are well defined, severe RSV disease may develop in otherwise healthy children. The pathogenesis of severe RSV disease is poorly defined.

In 2001, van den Hoogen et al. reported the isolation of a novel paramyxovirus, human metapneumovirus (HMPV) from children with respiratory tract disease (2). HMPV has been identified worldwide (3–7) and appears to have a seasonal distribution (winter and spring) (8). Since the circulation of HMPV may overlap with that of RSV, simultaneous infection with both RSV and HMPV may contribute to severe disease. Greensill et al. (9) reported that 70% of RSV-infected children who required admission to the Pediatric Intensive Care Unit (PICU) in Liverpool, U.K. were co-infected with HMPV.

We sought to determine whether infection with HMPV was associated with the severity of RSV disease. We determined the frequency of HMPV infection in children with either mild or severe RSV disease. Disease severity was assessed by both the disposition (PICU vs. non-PICU) and by a clinical severity score.

## The Study

As part of an ongoing epidemiologic study of viral respiratory infections in children, we collected all the RSV direct fluorescent antibody (DFA)–positive respiratory specimens from the Clinical Virology Laboratory at Yale-New Haven Hospital from November 1, 2001, to October 31, 2002. All respiratory specimens were also screened by DFA for parainfluenza viruses 1–3, influenza A and B viruses, and adenoviruses (10). Any RSV-positive patient who also tested positive for one of the viruses listed above was excluded from the study. All RSV-positive children admitted to PICU during this yearlong period were identified. Because the peak time of infection with RSV and with HMPV may differ, each RSV-positive child from PICU was matched by date of diagnosis with a child with mild RSV disease. All RSV-positive children who were not admitted to PICU and diagnosed within 2 days of the diagnosis of the PICU-admitted child were identified. Of these, the child whose age most closely matched the age of the PICU-admitted child was selected. If no child diagnosed with RSV was identified within 2 days of the PICU-admitted child, the child with the closest date of diagnosis was identified and matched to the PICU-admitted patient.

RNA extraction and reverse transcription–polymerase chain reaction (RT-PCR) were performed as previously described (4). Primers used for the amplification of the RSV L gene were as follows: forward primer 5´-GGTAGAATCTACATATCCTTACCTAAGTG-3´ (genome location 14881–14909, [reference strain RSV A Melbourne, Australia/2/’61] GenBank accession no. M74568), reverse primer 5´-CGAGATATTAGTTTTTGACAC-3´ (genome location 15190–15210, GenBank accession no. M74568). The HMPV forward primer, 5´-GCGCGTTCTGAGGACAGGTTGG-3´ (HMPV genome location 3163-3180, GenBank accession no. AF371367, G/C clamps are underlined) and reverse primer, 5´-GCGCTCAAGCCGGATGGTTTTGG-3´ (3425–3444, GenBank accession no. AF371367, G/C clamps are underlined) used for the amplification of the HMPV F gene were based on regions of the F gene conserved in New Haven, Netherlands, and Australian strains (4). PCR reactions were performed by using HotStar (Qiagen, Inc., Valencia, CA) and amplification cycles were as follows: 95°C for 15 min followed by 35 cycles of 94°C for 1 min, 55°C for 1 min, and 72°C for 1 min, and a final 10-min cycle at 72°C. Each set of reactions contained appropriate negative (water) and positive control samples for the RT (HMPV-positive nasal wash) and the PCR (HMPV cDNA) steps.

A clinical severity score (CSS) was adapted from the severity score described by Martinello et al. (11). Two points were assigned if the patient required positive-pressure ventilatory support during the illness, and one point was assigned for each of the following: hospital admission, hospitalization for >5 days, oxygen saturation <87% (at least one measurement), and any use of supplemental oxygen. Therefore, CSS ranged from 0 to 6. Medical records from all patients were abstracted and scored by a reviewer who did not know the patient’s HMPV status.

On the basis of the published literature (9), we expected the frequency of RSV/HMPV co-infection in the PICU patients to be approximately 70%, and in the non-PICU patients to be closer to the DFA-negative–HMPV-positive infection rate of 6.4% found in our population (DFA-negative refers to samples that tested negative for RSV, parainfluenza viruses, influenza viruses, and adenovirus) (4). Power calculations were performed by using PASS 2002 (J. Hintze, NCSS and PASS, Number Cruncher Statistical Systems, Kaysville, Utah). By using a power of 90% and a of 0.05, a sample of 12 patients in each group would be sufficient to support these findings (70% vs. 6.4%). By using the same power calculations, 23 patients in each group would provide adequate power to show as little as a 45% difference in proportions with co-infection between the two groups. Comparisons were made by using the chi-square test and Wilcoxon rank sum tests, as appropriate (Table). Exact 95% confidence intervals were calculated in SAS V8.2 (SAS Institute, Cary, NC).

**Table:** Comparison between the group of children admitted to PICU with severe RSV disease and the group of children with mild RSV disease

Characteristic	PICU^a^	Non PICU^a^	Statistical comparison (p value)
Median age (range)	7 wk (2 wk–21 mo)	54 wk (10 d–4 y)	0.025^b^
Prematurity (%)	5/23 (21.7)	3/23 (13.0)	> 0.1^c^
RSV^d^ PCR/DFA^e^ (%)	21/23 (91.3)	23/23 (100)	0.244^c^
Hospitalized (%)	23/23 (100)	8/23 (34.8)	0.004^c^
Median CSS^f^ (range)	5 (3–6)	1 (0–4)	< 0.001^b^
PPV^g^ (%)	17/23 (73.9)	0/23 (0)	ND^h^
RSV/HMPV^i^ co-infection	0/23 (0)	0/23 (0)	1.0

^a^PICU, pediatric intensive care unit. ^b^χ2 test. ^c^Wilcoxon rank sum test. ^d^RSV, respiratory syncytial virus. ^e^DFA, direct fluorescent antibody screen. ^f^CCS, clinical severity score. ^g^PPV, positive pressure ventilation. ^h^ND, non-comparable because patients requiring PPV are admitted to the PICU. ^i^HMPV, human metapneumovirus.

Twenty-three RSV DFA-positive patients were admitted to PICU during the study period, and 23 matched patients were identified. Demographic and clinical information were obtained for each patient from the medical record. Of the children admitted to PICU, 7 (30.4%) of 23 had a known predisposing risk factor for severe RSV disease. All the PICU-admitted children had CSS >3 and most (16 [70.0%] of 23) had a CSS >5. Eighteen (78.2%) of 23 PICU patients were hospitalized for >5 days. None of the RSV-positive patients admitted to the PICU tested positive for HMPV by RT-PCR.

Children with mild RSV disease were initially seen in the emergency department, and according to their severity of illness, either were discharged or were admitted to the pediatric ward. Eight patients (34.8%) of the 23 mild RSV disease group were admitted to the hospital, although only 4 (17.4%) of these children were hospitalized for >5 days. The CSS range for this group was 0–4. Most patients (14 [61.0%] of 23) had a CSS of <3. None of these patients were positive for HMPV by RT-PCR.

Statistically significant differences between the PICU group and the mild disease group were observed in admission age (median age 7 weeks vs. 54 weeks, p = 0.025), hospital admission rate (23/23 vs. 8/23, p = 0.004) and CSS (median CSS 5 vs. 1, p = 8 x 10^–12^) (Table). Positive pressure ventilation was required by 17 (73.9%) of 23 PICU patients and 10 of these 17 patients needed it for >5 days. None of the patients screened in either group of RSV-DFA positive patients had evidence of HMPV infection (p = 1.0). To ensure that the methods for RT-PCR were adequate, we performed RT-PCR for RSV for each patient’s respiratory specimen. Overall, 44 (95.7%) of 46 of children had a positive RT-PCR test for RSV.

## Conclusions

The possibility that HMPV plays a role in the pathogenesis of infections with other respiratory viruses is not known. The importance of identifying HMPV in persons with SARS remains to be explained (5,12). Greensill et al. observed a 70% co-infection rate with HMPV and RSV and a 90% co-infection rate among intubated infants with HMPV and RSV admitted to their PICU (9). Although Greensill et al. did not include an appropriate control group in their study, these findings suggest that co-infection with both HMPV and RSV is common and that together the two viruses may contribute to increase the severity of disease.

We did not observe HMPV infection in children with either mild or severe RSV disease. Our findings cannot be explained by the absence of HMPV in Connecticut. From November 2001 to April 2002 (the period of time when all of the RSV-positive PICU-admitted children were identified), HMPV was detected in 11% of 446 patients who tested negative by DFA for RSV, parainfluenza viruses, influenza virus, and adenovirus (13). Furthermore, we matched children with mild RSV disease to children with severe RSV disease by date of diagnosis to eliminate the possibility that the temporal distribution of the viruses might influence our results.

The potential difference in the sensitivity of the screening tests used by Greensill et al. and our group likely does not account for the differences in the observed rates of co-infection. We used a similar RT-PCR–based approach. We are confident that our methods to detect HMPV are both sensitive and specific (4,13). The observed rate of HMPV in respiratory specimens in our previous study (8.1%) (13) is comparable to rates observed elsewhere (6,14). Furthermore, in our previous studies, we have used sequence analysis of RT-PCR amplicons to confirm the identification of HMPV (4,13). An increased prevalence of HMPV in Liverpool, UK, may account for the high rate of RSV/HMPV co-infection observed by Greensill et al., although no data at this point support this hypothesis.

Our relatively small sample size limited the power of our analysis. However, on the basis of the sample size calculations with 90% power, our patient numbers were sufficient to detect a difference of >45% above the rate of HMPV infection in the non-PICU group (6.4%). Nonetheless, our results demonstrate that the rate of co-infection is low (0% of 23 patients, 95% confidence interval 0%–14.8%). Other studies also support our findings that the frequency of co-infection with HMPV and RSV is rare (7,14)

The basis of the pathogenesis of severe RSV disease is multifactorial. Since severe RSV disease may develop in apparently healthy children, known host risk factors cannot completely account for instances of severe illness. Preexisting or maternally acquired immunity, innate immunity, viral factors and genotypes and environment all likely contribute to disease pathogenesis. Although we did not detect co-infection, HMPV may worsen RSV disease in a small percentage of infants. Nonetheless, HMPV most likely does not play an important role in the severity of RSV disease in the population.
